# Sustained lung inflation in the delivery room in preterm infants at high risk of respiratory distress syndrome (SLI STUDY): study protocol for a randomized controlled trial

**DOI:** 10.1186/1745-6215-14-67

**Published:** 2013-03-08

**Authors:** Carlo Dani, Gianluca Lista, Simone Pratesi, Luca Boni, Massimo Agosti, Paolo Biban, Antonio Del Vecchio, Diego Gazzolo, Camilla Gizzi, Rosario Magaldi, Hubert Messner, Fabio Mosca, Fabrizio Sandri, Fabio Scopesi, Daniele Trevisanuto, Giovanni Vento

**Affiliations:** 1 Department of Surgical and Medical Critical Care, Section of Neonatology, Careggi University Hospital, Viale Morgagni 85, Florence, 50141, Italy; 2 Division of Neonatology, ‘VBuzzi’ Children’s Hospital, Via Castelvetro. 32, Milan, 20154, Italy; 3 Clinical Trials Coordinating Center, IstitutoToscanoTumori, Florence; Department of Human Pathology and Oncology, University of Florence, Viale Morgagni 85, Florence, 50141, Italy; 4 Maternal and Child Health Department, Del Ponte Hospital, A.O. Di Circolo Fondazione Macchi, Via Del Ponte, 19, Varese, 21100, Italy; 5 Department of Pediatrics, Pediatric and Neonatal intensive Care Unit, Azienda Ospedaliera Universitaria Integrata, Oiazzale Stefani, 1, Verona, 37126, Italy; 6 Division of Neonatology, Neonatal Intensive Care Unit, Di Venere Hospital, Bari, Via Ospedale di Venere 150, Bari, 70012, Italy; 7 Department of Maternal Fetal and Neonatal Medicine, C. Arrigo Children’s Hospital, Via Santa Caterina da Siena, 30, Alessandria, 15121, Italy; 8 Division of Neonatology, S. Giovanni Calibita Hospital Fatebenefratelli, Piazza Fatebenefratelli 1, 00186, Rome, Italy; 9 Division of Neonatology, Neonatal Intensive Care Unit, Ospedali Riuniti, Via degli Aviatori, 1, Foggia, 71122, Italy; 10 Neonatal Intensive Care Unit, Ospedale Regionale, Via L. Böhler, 5, Bolzano, 39100, Italy; 11 Department of Mother and Infant Science, Neonatal Intensive Care Unit, Fondazione IRCCS ‘Ca’ Granda’Ospedale Maggiore Policlinico, Via Commenda, 12, Milan, 20122, Italy; 12 Maternal and Pediatrics Department, Maggiore Hospital, Largo Nigrisoli, 2, Bologna, 40100, Italy; 13 Department of Neonatology Obstetrics and Neuroscience, G. Gaslini Children’s University Hospital, Via Gerolamo Gaslini, 5, Genoa, 16148, Italy; 14 Department of Pediatrics, Azienda Ospedaliera of Padua, Viale Giustiniani, 2, Padua, 35100, Italy; 15 Division of Neonatology, Catholic University of Rome, Largo Agostino Gemelli 8, Rome, 00168, Italy; 16 Division of Neonatology, Careggi University Hospital, University of Florence School of Medicine, Viale Morgagni, 85, Florence, 50141, Italy

**Keywords:** Sustained lung inflation, Preterm infants, Resuscitation, Delivery room, Mechanical ventilation

## Abstract

**Background:**

Some studies have suggested that the early sustained lung inflation (SLI) procedure is effective in decreasing the need for mechanical ventilation (MV) and improving respiratory outcome in preterm infants. We planned the present randomized controlled trial to confirm or refute these findings.

**Methods/Design:**

In this study, 276 infants born at 25^+0^ to 28^+6^ weeks’ gestation at high risk of respiratory distress syndrome (RDS) will be randomized to receive the SLI maneuver (25 cmH_2_O for 15 seconds) followed by nasal continuous positive airway pressure (NCPAP) or NCPAP alone in the delivery room. SLI and NCPAP will be delivered using a neonatal mask and a T-piece ventilator.

The primary endpoint is the need for MV in the first 72 hours of life. The secondary endpoints include the need and duration of respiratory support (NCPAP, MV and surfactant), and the occurrence of bronchopulmonary dysplasia (BPD).

**Trial registration:**

Trial registration number: NCT01440868

## Background

The pathogenesis of bronchopulmonary dysplasia (BPD) in preterm infants is multifactorial, but the role of ventilator-induced lung injury (VILI) is very important [1]. Although the respiratory support of preterm infants with respiratory distress syndrome (RDS) has improved and new modes of mechanical ventilation (MV) have been developed, the occurrence of BPD has remained substantially unchanged. Nevertheless, one aspect of respiratory care in preterm infants which has not yet been thoroughly investigated is respiratory assistance in the delivery room immediately after birth. In fact, lung protection should start in the delivery room where, from the first breaths, the preterm infant can be helped to clear the lung fluid; to recruit alveolar spaces facilitating the formation of the functional residual capacity (FRC); to protect the lung by avoiding large tidal volume; to avoid the continuous ‘opening and closing’ of the alveoli by delivering positive end-expiratory pressure (PEEP); and to verify the need for surfactant replacement [2,3].

Recently, some studies have investigated the effectiveness of the early sustained lung inflation (SLI) procedure in preterm infants for prevention of MV. This strategy would permit lung recruitment immediately after birth through delivery of brief peak pressure to the infant airways via a nasopharyngeal tube or mask allowing preterm infants to achieve FRC. To avoid lung collapse at the end of the expiration, the effect of PEEP is crucial. SLI and PEEP seem to have an additive effect on adequate FRC formation by permitting optimal gas exchange, improving lung mechanics and reducing the need for respiratory support.

This technique has been proven to be more effective than intermittent mandatory ventilation (IMV) in improving FRC in the asphyxiated term newborn [4,5]. Lindner *et al*. treated preterm infants by using SLI in the delivery room without a significant decrease in the MV rate or adverse effects in comparison with treatment with nasal-intermittent mandatory ventilation (NIMV) noted [6]. On the other hand, te Pas *et al*. treated preterm infants with repeatable SLI maneuvers and found a decrease in the need for MV at 72 hours of life and BPD in comparison with treatment in the delivery room with a self-inflating bag [7]. These results are in agreement with the findings of a recent paper from the same group, which demonstrates the synergistic effect of SLI and following subsequent treatment with PEEP (delivered by nasal continuous airway pressure (NCPAP)) in achieving and maintaining a FRC improvement in an animal model [8]. Moreover, Lista*et al*. recently reported that SLI followed by the delivery of NCPAP is effective in reducing the need for MV and the occurrence of bronchopulmonary dysplasia in survivors compared to NCPAP alone [9].

We therefore intend to compare the application of SLI followed by NCPAP with NCPAP alone in the delivery room to evaluate its effectiveness in decreasing the need for MV and improving respiratory outcome in preterm infants at risk for RDS.

## Methods/Design

### Aims

The primary aim of this study is to compare the need for MV in the first 72 hours of life (excluding the transient tracheal intubation performed for surfactant administration) in infants born at 25^+0^ to 28^+6^ weeks’ gestation who received the SLI maneuver in the delivery room or not.

### Study design

This will be an unblinded multicenter randomized trial of SLI versus noSLI in infants born at 25^+0^ to 28^+6^ weeks’ gestation.

### Inclusion criteria

Inborn infants satisfying the following inclusion criteria will be eligible to participate in the study:

1. Born at 25^+0^ to 28^+6^ weeks’ gestation (and)

2. High risk of RDS (and)

3. Parental consent has been obtained.

### Exclusion criteria

1. Presence of major congenital malformations.

2. Fetal hydrops.

3. Inherited disorders of metabolism.

### Primary outcome measure

The primary endpoint of this study will be the need for MV within the first 72 hours of life. Therefore, we will consider SLI a success if MV is not required and a failure if the infant needs MV. The need for MV is defined later under ‘Criteria for starting MV’.

We have chosen the need for MV during the first 72 hours of life as our primary outcome measure to maintain similarity with the main previous trial conducted on this issue [7].

### Secondary outcome measures

1. Need for conventional or high frequency modes of ventilation in the first 3 hours of life.

2. Highest FiO_2_ and oxygentherapy duration.

3. Duration of NCPAP, need and duration of bi-level nasal continuous positive airway pressure (BiPAP) and NIMV.

4. Need and duration of conventional MV (synchronized intermittent mechanical ventilation (SIMV), synchronized intermittent positive pressure ventilation (SIPPV), pressure support ventilation (PSV) with or without with volume guarantee (VG)) or high frequency ventilation (HFV).

5. Duration of hospitalization.

6. Highest mean airway pressure (MAP) during MV.

7. Need and number of doses of surfactant.

8. Occurrence of mild, moderate and severe bronchopulmonary dysplasia (BPD) [10].

9. Death.

10. Death or severe BPD.

### Other collected data

The following data will be recorded for each infant: gestational age (GA), birth weight (BW), sex, Apgar score at 5 minutes, antenatal steroid treatment, CRIB II score [11], occurrence of pneumothorax, patent ductus arteriosus (PDA) and need of surgical closure, grade 3 to 4 intraventricular hemorrhage (IVH) [12], periventricular leukoma-lacia (PVL) [13], grade >2 retinopathy of prematurity (ROPand ROP requiring surgery [14], necrotizing enterocolitis (NEC) [15], sepsis [16], length of stay in neonatal intensive care unit (NICU) and hospital, and mortality. Patients will be discharged from the NICU to a lower level of care when they no longer need respiratory assistance other than oxygentherapy and central venous catheters.

### Sample size

We hypothesize that SLI maneuver might decrease the need for MV during the first 72 hours of life from 35% to 20%. We calculated that 138 newborns must be enrolled in each group to detect this difference as statistically significant with 80% power at a level of 0.05.

### Recruitment

Written and oral information will, whenever possible, be offered to parents prior to the birth of their child if there is a risk that the mother will have a preterm delivery and the infant is likely to be eligible. Informed written consent will be signed by both the parents and sufficient time will be allowed for consent. Non-Italian speaking parents will only be asked for their consent if an adult interpreter is available. Trust interpreter and link worker services will be used to support involvement of participants whose first language is not Italian. A senior investigator will be available at all times to discuss concerns raised by parents or clinicians during the course of the trial.

A monthly accrual report of the study will be sent to participating centers.

### Randomization

Infants at each unit will be assigned to a block (1st block: gestational age from 25^+0^ to 26^+6^ weeks; 2nd block: gestational age from 27^+0^ to 28^+6^ weeks) and randomly assigned to a treatment group in 1:1 ratio using automatically generated sealed opaque envelopes, which will be prepared at Careggi University Hospital, Florence, Italy, and then distributed to participating centers. Block size will be concealed to investigators to ensure treatment balance between the two arms of the study.

### Blinding

The study will not be blinded and the staff performing the study will also care for the infants later on. However, the decision to start MV will be made by clinicians other than the investigators involved in patient care and researchers assessing study end-points will be blinded to the nature of the study treatments.

To minimize bias, strict criteria and definitions will be maintained during the trial.

### SLI maneuver

Infants in the SLI group will undergo the following approach. After oropharyngeal and nasal suctioning, pre-ssure-controlled (25 cmH_2_O) inflation will be sustained for 15 seconds, using a neonatal mask and a T-piece ventilator, followed by the delivery of 5 cmH_2_O NCPAP. Patients will be observed for the following 6 to 10 seconds to evaluate their cardio-respiratory function. If respiratory failure persists (that is, apnea and gasping) and/or the heart rate is >60 and <100 bpm despite NCPAP, the SLI maneuver (again 25 cmH_2_O for 15 seconds) will be repeated. If the heart rate remains >60 and <100 bpm after the second SLI maneuver, the infant will be resuscitated following the current guidelines of the American Academy of Pediatrics (AAP) [17]. Moreover, if the heart rate remains <60 bpm the infant will be resuscitated following the current guidelines of the AAP [17], and a second SLI maneuver will be repeated when the heart rate increases to >60 and <100 bpm. Infants in the control group will be treated with delivery of 5 cmH_2_O NCPAP and will be assisted following the guidelines of the AAP [18].

All enrolled infants will be transferred to the NICU in NCPAP (PEEP at 5 cmH_2_O).

Neonatal care will be started with FiO_2_ of 0.21 to 0.40 in both the groups, in agreement with the local protocols. Respiratory support in the delivery room will be given using the same T-piece ventilator (Neopuff Infant T-Piece Resuscitator, Fisher & Paykel, Auckland, New Zealand), which is a pressure-limited mechanical device that supplies consistent peak inspiratory pressure (PIP) and PEEP, and is capable of delivering sustained inflation [7]. To avoid pressure leakage, a neonatal mask of appropriate size which adequately covers both the mouth and nostrils of infants will be used. The flow rate will be set at 8 to 10 l/min without changes during the resuscitation.

### Criteria for starting MV

In the delivery room, infants will be started on MV if they have not reached the goal of 70% SpO_2_ by 5 minutes [19] and 85% by 10 minutes of life [17] with a heart rate >100 bpm, despite NCPAP at 5 cmH_2_O. In the NICU, infants will be started on MV when the pH is <7.20 with PaCO_2_>65 mmHg, PaO_2_<50 mmHg with FiO_2_ ≥0.50 after surfactant treatment, or if infants have frequent episodes of apnea (>4 episodes in 1 hour or >2 episodes requiring bag-and-mask ventilation), despite adequate NCPAP (5 to 7 cmH_2_O) delivery and oxygenation. MV will be set to maintain a PaCO_2_ of 55 to 65 mmHg and 88 to 95% SpO_2_.

### Other aspects of respiratory support

To maintain an adequate SpO_2,_ infants with an FiO_2_ ≥0.40 will be treated with surfactant (200 mg/kg Curosurf, ChiesiPharmaceuticals, Parma, Italy) followed by the reinstitution of NCPAP as soon as vital signs are satisfactory (intubation-surfactant-extubation (INSURE) strategy). All infants who will need MV will be treated with surfactant and will then be gradually weaned from it. Additional doses of surfactant (100 mg/kg) will be given to infants, also through the INSURE strategy, at the discretion of the attending neonatologist.

Infants will be extubated, after a loading dose of caffeine citrate (20 mg/kg), when they meet all of the following criteria: FiO_2_<0.40, PaCO_2_<65 mmHg with a pH >7.20, MAP <7 cmH_2_O, hemodynamic stability and the absence of clinically significant patent ductus arteriosus.

### Data collection

All collected data will be obtained from the clinical records. They will be recorded on electronic data sheets designed for this study. Data will be entered by the local principal investigator on a web-based electronic case record form. Access to the form will be password protected and participants will be identified by trial number only.

Clinical information will be collected at the following times:

1. 1.At trial entry: eligibility, background information and randomization

2. 2.Following randomization: all data above listed in ‘Primary outcome measure’, ‘Secondary outcome measures’ and ‘Other collected data’ sections.

Further information will be collected on expected serious adverse events (SAEs).

### Statistical analysis

The primary efficacy analysis will be conducted on an intention-to-treat basis. Clinical characteristics of infants in the SLI and control groups will be described using mean values and SD, median value and range, or rate and percentage. Univariate statistical analysis will be performed using the Student’st-test for parametric continuous variables, the Wilcoxon rank-sum test for non-parametric continuous variables and the chi-square test for categorical variables. A *P*<0.05 will be considered statistically significant. SLI treatment and clinical characteristics which are most likely associated with the need for MV (gestational age, birth weight, antenatal steroids, CRIB score, INSURE procedure and hospital of birth) will be included in multiple logistic regression analysis to assess their independent role in predicting SLI success or failure. Effect estimates will be expressed as relative risk (RR) with profile likelihood-based 95% confidence limits.

An interim analysis is planned when 100 infants are enrolled (50 in each arm).

### Duration of study

In this study, 272 infants will be recruited. The trial will terminate when the last recruited infant is discharged from hospital, or dies.

## Quality control and quality assurance procedures

### Compliance to protocol

Compliance will be defined as full adherence to protocol. Compliance with the protocol will be ensured by a number of procedures as described below.

### Site set-up

Local principal investigators participated in preparatory meetings in which details of the study protocol, data collectionand SLI procedure were accurately discussed. All centers received detailed written instruction on web-based recording data and, to resolve possible difficulties, it will be possible to contact the Clinical Trials Coordinating Center (LB). Moreover, it has been ascertained that the SLI procedure is equally made in all participating centers, to avoid expensive visits for training at each site due to the limited resources available for this study.

### Data processing and monitoring

All study data will be:

1. Screened for out-of-range data, with cross-checks for conflicting data within and between data collection forms by a data manager.

2. Referred back to the relevant center for clarification in the event of missing items or uncertainty.

3. Reviewed by the Chief Investigators (CD, GL) and trial statistician, and they will review the results generated for logic,patterns andproblems Outlier data will be investigated.

### Safety

Safety end-points will include incidence, severity and causality of reported SAEs, namely changes in occurrence of the expected common prematurity complications and clinical laboratory test assessments, and the development of unexpected SAEs in this high risk population. All SAEs will be followed until satisfactory resolution or until the investigator responsible for the care of the participant deems the event to be chronic or the patient to be stable.

All expected and unexpected SAEs, whether or not they are attributable to the study intervention, will be reviewed by the local principal investigators to determine if there is a reasonable suspected causal relationship with the intervention. If the relationship is reasonable, SAEs will be reported to the Chief Investigators who will then report them to the Ethics Committee and inform all other investigators to guarantee the safety of the participants.

## Trial status

The Local Ethics Committee approved the study and the trial is currently recruiting study subjects.

## Abbreviations

AAP: American Academy of Pediatrics; BDP: bronchopulmonary dysplasia; BiPAP: bi-level nasal continuous positive airway pressure; BPD: bronchopulmonary dysplasia; BW: birth weight; FRC: functional residual capacity; GA: gestational age; HFV: high frequency ventilation; IMV: intermittent mandatory ventilation; INSURE: intubation-surfactant-extubation; IVH: intraventricular hemorrhage; MAP: mean airway pressure; MV: mechanical ventilation; NCPAP: nasal continuous positive airway pressure; NEC: necrotizing enterocolitis; NICU: neonatal intensive care unit; NIMV: nasal intermittent mandatory ventilation; PDA: patent ductus arteriosus; PEEP: positive end-expiratory pressure; PIP: peak inspiratory pressure; PSV: pressure support ventilation; PVL: periventricular leukomalacia; RDS: respiratory distress syndrome; ROP: retinopathy of prematurity; RR: relative risk; SAE: serious adverse event; SIMV: synchronized intermittent mechanical ventilation; SIPPV: synchronized intermittent positive pressure ventilation; SLI: sustained lung inflation; VG: volume guarantee; VILI: ventilator-induced lung injury

## Competing interests

The authors declare that they have no competing interests.

## Authors’ contributions

CD, GL, SP, MA, PB, ADV, DG, CG, RM, HM, FM, FS, FS, DT and GV have made substantial contributions to the conception and design of the study protocol, and have given final approval of the version to be published. LB prepared electronic data sheets, wasresponsible for the web-based electronic case record form and has given final approval of the version to be published. All authors read and approved the final manuscript.
